# Editorial: How Can Genomic Biobanks Provide the Bridge for Implementation of Effective Clinical Therapy?

**DOI:** 10.3389/fpubh.2020.581490

**Published:** 2020-09-22

**Authors:** Ronald M. Przygodzki

**Affiliations:** Office of Research & Development, Washington, DC, United States

**Keywords:** biobanking & biorepositories, genomic, ethic, database, standards, policy

While the era of isolated large medical record databases, unsystematic access to and performance of genetic testing and rising healthcare costs are at hand, we have an opportunity to develop solutions and tools that could unify and harmonize these efforts. Through the implementation of the US Department of Veterans Affairs Million Veterans Program (MVP), with over 700,000 voluntarily consented participants allowing blood sampling for a variety of prospective genomic testing, access to medical records, and survey materials, we have unearthed multitudes of biobanking-related challenges. Through intellectual partnerships on committees and panels, we share similar discoveries, and seek common solutions. We turned to *Frontiers* in an effort to help all interested in establishing a biobank to share some of their challenges, possible goals and standards for unification that might be taken into consideration globally if we are to develop systems that are truly comparable, translatable, actionable, and most importantly, take into account the ethics, safety, and individuality of the partnering patient.

We have compiled a collection of unique and informative articles that will engage and inform the readers, and provide guidance necessary for success in their biobanking venture. While they span a variety of important aspects and focus on specific, limited challenges, each deliver much food for thought on how these groups have addressed their situation. They demonstrate the breadth of consideration needed for long-term success.

We have an article that looks at Prictor et al.. These authors share with us the aspects that lead to bias in recruitment, and how to recognize and consider the barriers that hinder participation, including location, literacy, language, age, and culture of the individual. The granularity available within a dynamic consent to a participant can address some such aspects while improving engagement.

The collection has an article addressing (Wang et al.). These authors express the need for accurate phenotyping to improve and grow precision medicine diagnostics, especially in research. They note that while numerous computational phenotyping algorithms are published, the true culprit hindering adequate phenotyping is incomplete data within one single data source (data fragmentation). They demonstrate the impact of diverse data sources on two published computational phenotyping algorithms used within their own institutional database, that being rheumatoid arthritis and type 2 diabetes mellitus. The impact of data fragmentation on these algorithms is discussed and what further causes should be considered in possibly mitigating such situations. These findings can likewise have an impact on other nationally recognized phenotyping tools.

We have a group informing us on Hathcock et al.. These authors, consenting participants into their Mayo Clinic biobank, have found striking characteristics within their population that are suggestive of increased willingness to participate. An inverse of sex and age appear to be factors; young males are less likely to participate than young females, whereas older females are less likely to participate than older males. Educational level of the participants plays a positive factor, as does certain races. Specific demographics likewise play a role in follow up research. These findings are useful to be aware of, and possibly find other solutions to bolster participation.

We have an article sharing (Cicek and Olson). The authors note the need for banks to help improve population health, share new knowledge in treating disease, and how individual participants augment the large collective in such works. They provide an overview of important considerations for biorepository establishment, operational systems, safeguards, and sustainability approaches that support biospecimen collection and storage across multiple biobanks.

While the development of biobanks might be considered a collection of items, an outcome of improved healthcare is typically one of the major benchmarks for successful use of such a bank. Unfortunately, health inequities in genomic medicine are present, and require a need for understanding of Caron et al.. The authors, discussing their experience with indigenous populations within Canada and New Zealand, are certainly applicable and extendable to underrepresented minorities and groups worldwide. They provide rationale for approaching and informing such populations, which can lead to increased accrual and understanding of genomic variants unique to these peoples.

Finally, we have an example of biobank use that might not be immediately apparent, that being use of readily available data in Cheng et al.. Central to this study, the authors have used genomic findings from the UK Biobank, data on bone mineral density in individuals and genomic data of gut microbiome and through polygenic risk score development were able to identify a novel association. Importantly, all of these data are high quality, and essentially performed on the datasets alone making the discoveries faster and much more compact in time. Such studies allow the investigators the freedom to easily and cost-effectively seek answers to clinically relevant questions. It can also allows one to synthesize strong hypotheses that can truly effectively answer seemingly disparate assumptions with greater statistical rigor through secondary studies.

On behalf of my co-topic editors Drs. Kilbourne, Johansen-Taber, and Mahfouz, we invite you to enjoy the collection. We ask to please continue to submit manuscripts to further aid the biobanking community in the ever-evolving quest for the perfect, all encompassing and ethical biobank.

## Author Contributions

The author confirms being the sole contributor of this work and has approved it for publication.

## Conflict of Interest

The author declares that the research was conducted in the absence of any commercial or financial relationships that could be construed as a potential conflict of interest.

